# The role of icodextrin in peritoneal dialysis: protocol for a systematic review and meta-analysis

**DOI:** 10.1186/s13643-019-0959-y

**Published:** 2019-01-30

**Authors:** Monika Becker, Stefanie Bühn, Jessica Breuing, Catherine A. Firanek, Simone Hess, Hisanori Nariai, Mark R. Marshall, James A. Sloand, Qiang Yao, Käthe Goossen, Dawid Pieper

**Affiliations:** 1 Institute for Research in Operative Medicine (IFOM), Department for Evidence Based Health Service Research, Faculty of Health, Department of Medicine, Witten/Herdecke University, Ostmerheimer Str. 200, Building 38, 51109 Cologne, Germany; 2 Medical Affairs, Baxter Healthcare International, 1 Baxter Pkwy, Deerfield, IL 60015 USA; 3 Baxter Co. Ltd., Toranomon Hills Mori Tower 20F, 23-1, Toranomon 1-Chome, Minato-ku, Tokyo, 105-6320 Japan; 4 Medical Affairs, Baxter Healthcare (Asia) Pte Ltd., 150 Beach Road, #30-01/08 Gateway West, Singapore, 189720 Singapore; 5 School of Medicine, Faculty of Medical and Health Sciences, University of Auckland, Private Bag 92019, Auckland, 1142 New Zealand; 6 Department of Renal Medicine, Counties Manukau District Health Board, Private Bag 93311, Auckland, 1640 New Zealand; 7 Medical Affairs, Baxter (China) Investment Co., Ltd., 12F Century Business Plaza, 989 Changle Road, Shanghai, 200031 China

**Keywords:** Icodextrin, Peritoneal dialysis, Glucose-based solutions, Systematic review, Meta-analysis

## Abstract

**Background:**

Previous meta-analyses have found several advantages of icodextrin compared with glucose in the application of peritoneal dialysis (PD), such as an improvement of peritoneal ultrafiltration during the long dwell and a reduction in episodes of uncontrolled fluid overload. However, the effect of icodextrin on patient-relevant outcomes remains unclear. This review aims to evaluate the benefits and harms of icodextrin in comparison with conventional glucose PD solution in patients with end-stage kidney disease receiving PD.

**Methods:**

Randomized controlled trials of icodextrin comparing with conventional glucose solution in patients with end-stage kidney disease who received PD will be deemed eligible. We will conduct systematic searches in MEDLINE, EMBASE, CENTRAL, Ichushi-Web, Chinese and Japanese databases, and in clinical trials registries (ClinicalTrials.gov, International Clinical Trials Registry Platform Search Portal (ICTRP), EU Clinical Trials Register, Japan Registries Network (JPRN), China’s Clinical Trial Registry (ChiCTR)). Furthermore, we will check conference proceedings and search references from relevant studies manually. Relevant pharmaceutical companies, authors, and experts will be contacted in an effort to identify further studies. We will not apply any limitations regarding language, publication status, and publication date when searching for eligible studies. The selection of studies, data extraction, and risk of bias assessment will be carried out by two independent reviewers. Data synthesis will be performed using RevMan 5 software with either a fixed effects model or random-effects model, depending on the presence of heterogeneity. For the assessment of statistical heterogeneity, *I*^2^ will be calculated. Sources of clinical heterogeneity will be evaluated through subgroup analyses. If there are ten or more studies included in the meta-analysis, we will investigate the publication bias using funnel plots and Egger’s test. The quality of the body of evidence will be assessed using the Grading of Recommendations Assessment, Development and Evaluation (GRADE) system.

**Discussion:**

We assume that our systematic review will be more comprehensive compared to those published previously due to contacting the relevant pharmaceutical companies and a systematic search of published and unpublished non-English studies from China, Taiwan, and Japan.

**Systematic review registration:**

PROSPERO CRD42018096951

## Introduction

### Rationale

Peritoneal dialysis (PD) is a major global modality for renal replacement therapy. There are approximately 200,000 PD patients worldwide [1, 2]. The use of icodextrin over time has increased since its launch in the mid-1990s, and as of 2013, over 30,000 patients globally were receiving icodextrin treatment [3].

Glucose is the most commonly used osmotic agent in PD and is included in the final manufactured product in variable concentrations to meet the different ultrafiltration needs of patients. However, glucose solutions degrade quickly in the peritoneum and have short-lived effect as an osmotic agent—longer dwells of such solutions can often result in net reabsorption of fluid from the dialysate into the patient, rather than the intended effect which is the other way around. Furthermore, glucose itself and especially its degradation products generated during manufacturing are toxic to the peritoneal membrane [4–6], resulting in inexorable peritoneal damage that functionally limits the effectiveness and longevity of PD therapy. Finally, these solutions lead to metabolic derangements such as hyperglycemia, hyperinsulinemia, and hyperlipidemia [7].

As an alternative to conventional glucose PD solutions, several non-glucose PD fluids have been developed. The most widely used is icodextrin, generally used in the concentration of 7.5% in this application. Icodextrin is a water-soluble glucose polymer and acts as a colloidal osmotic agent. Previous meta-analyses have found several advantages of icodextrin compared with glucose, such as an improvement of peritoneal ultrafiltration during the long dwell, especially in patients with high or high-average peritoneal transport status [8–10]. There is also evidence for a reduction in episodes of uncontrolled fluid overload [8, 9, 11]. Regarding the impact on peritoneal creatinine clearance, previous systematic reviews have shown different results [8–12]. The effect of icodextrin on patient-relevant outcomes, such as patient survival and hospitalization, is still unclear, maybe because of the frequently small study sizes and short follow-up durations.

As the authors of previous meta-analyses did not contact pharmaceutical companies, some published and unpublished studies might not be included in these analyses. The Cochrane review of Cho et al. comprised searches in the clinical trials registries *ClinicalTrials.gov* and *International Clinical Trials Registry Platform Search Portal* (ICTRP) [9]. Although the Cochrane review was recently updated [11], the rationale for our review remains unchanged. We believe that adding further registries, such as the *Japan Registries Network* (JPRN) and *China’s Clinical Trial Registry* (ChiCTR), and additional searches in Chinese and Japanese databases could lead to a more comprehensive review.

### Objectives

We aim to evaluate the benefits and harms of icodextrin in comparison with conventional glucose PD solution in patients with end-stage kidney disease receiving PD.

## Methods

### Eligibility criteria

We will include published and unpublished studies irrespective of their language, if they meet the following criteria.

#### Population

Adults and children with end-stage kidney disease who are receiving any type of PD (continuous ambulatory PD (CAPD), automated PD (APD)/continuous cyclic peritoneal dialysis (CCPD), (nocturnal) intermittent PD (IPD/NIPD), tidal peritoneal dialysis (TPD) or continuous flow peritoneal dialysis (CFPD)) will be considered for inclusion.

#### Intervention

The study group will include all patients who received PD with icodextrin.

#### Comparison

The control will include all patients who received PD with conventional glucose solution at any concentration.

#### Outcome

The outcomes are any patient-relevant and clinical outcomes listed in this protocol under the “Outcomes and prioritization” section.

#### Study designs

We will only include (quasi-) randomized controlled trials (RCTs) in our systematic review. Systematic reviews related to the topic will be retained to investigate their references for further eligible studies.

### Information sources

We will conduct a systematic literature search to identify all published and unpublished studies. The following databases will be searched for citations from inception to present: MEDLINE (via PubMed); EMBASE (via EMBASE); CENTRAL (via the Cochrane Library); Ichushi-Web; and Chinese databases as well as clinical trials registries (ClinicalTrials.gov, International Clinical Trials Registry Platform Search Portal (ICTRP), EU Clinical Trials Register, Japan Registries Network (JPRN), China’s Clinical Trial Registry (ChiCTR)); China National Knowledge Infrastructure (www.cnki.net); Chongqing VIP Information Co., Ltd., formerly known as Database Research Center under Chongqing Branch of Institute of Scientific & Technical Information of China (CB-ISTIC, www.wanfangdata.com.cn); HK Government Library (https://www.hkpl.gov.hk/en/e-resources/e-databases/keyword/e-database/all/1); HyRead Full text Database of Taiwan (http://www.hyread.com.tw/hyreadnew/); Ericdata Higher Education Knowledge Base (http://www.ericdata.com/); Taiwan Journal Papers Index System (http://readopac.ncl.edu.tw/nclJournal/index.htm); TAO Taiwan Academic Online (http://tao.wordpedia.com/); and Ariti library (http://www.airitilibrary.com/).

Additionally, we will check the conference proceedings of the American Society of Nephrology annual meetings, the European Nephrology Conferences, the World Congresses of Nephrology, and the congresses of the International Society for Peritoneal Dialysis for the past 25 years (1993–2018). We will search manually for additional studies by cross-checking the reference lists of all included primary studies and lists of relevant systematic reviews. Furthermore, we will contact the relevant pharmaceutical companies (Baxter Healthcare (http://www.baxter.com/), Terumo (http://www.terumo.com/)) in an effort to identify further studies. In addition, study authors and experts will be contacted for additional studies.

We will not apply any limitations regarding language, publication status, and publication date when searching for eligible studies.

### Search strategy

The search strategy will be developed by the research team in collaboration with an experienced librarian and checked by a referee according to the Peer Review of Electronic Search Strategies (PRESS) guideline [13]. A draft of the PubMed search strategy is presented as follows:

- (“Peritoneal Dialysis”[Mesh] OR “Peritoneal Dialysis, Continuous Ambulatory”[Mesh] OR peritoneal dialysis[tiab] OR PD[tiab] OR CAPD[tiab] OR CCPD[tiab] OR APD[tiab] OR IPD[tiab] OR NIPD[tiab] OR CFPD[tiab] OR TPD[tiab])
- AND (“icodextrin” [Supplementary Concept] OR icodextrin*[tiab] OR extraneal[tiab] OR nicopeliq[tiab] OR biocompatib*[tiab] OR glucose polymer[tiab] OR polyglucose[TIAB] OR maltose[TIAB] OR dextrin[TIAB] OR icodial[TIAB]).
- AND ((Randomized controlled trial [pt] OR controlled clinical trial [pt] OR randomized [tiab] OR randomised [tiab] OR placebo [tiab] OR randomly [tiab]) NOT (animals [mh] NOT humans [mh])).
- NOT (“Comment” [Publication Type] OR “Letter” [Publication Type] OR “Editorial” [Publication Type]).

### Data management

The search results will be uploaded and managed using Microsoft Excel. Duplicates will be removed manually. The search interfaces, dates, terms, and results will be documented for each database.

### Selection process

The title and abstract of each article will be screened and assessed against predefined inclusion criteria by two independent reviewers (MB and JB). Full texts of all potentially relevant articles will be assessed for inclusion by two reviewers (MB and JB) independently. Disagreements will be resolved through discussion and consensus or consulting a third reviewer (DP). The corresponding authors of eligible articles will be contacted for clarification where necessary. We will record the reasons for the exclusion and report the study selection process using the PRISMA flow diagram. A list of excluded studies will be provided.

### Data collection process

A standardized data extraction sheet will be designed and tested. Two reviewers (MB and KG) will independently extract data from the included studies. Any disagreements will be resolved through discussion and consensus or by involving a third reviewer (DP). Where necessary, studies will be translated before the assessment and data extraction.

### Data items

The following data will be collected:

- Study characteristics (design, sample size, duration of follow-up, number of patients randomized and included in the analysis, concentration of glucose in the control group)
- Patients’ characteristics: demographics (age, sex), relevant medical conditions (ASA score, cause of end-stage renal disease, peritoneal membrane transport characteristics as defined by the peritoneal equilibration test (PET), estimated (residual) glomerular filtration rate)
- Mode of PD
- Outcomes

In case outcome data are missing, we will contact the study authors and request the data.

### Outcomes and prioritization

The primary outcomes will be:

- Patient survival (number of patients alive at study completion)
- Technique survival (number of patients remaining on PD at study completion)
- Quality of life (any questionnaire used by the authors)
- Ultrafiltration (UF) (net UF/net UF change/total UF/UF efficiency ratio)

Secondary outcomes will be:

- Serious adverse events (e.g., uncontrolled fluid overload, peritonitis)
- Total adverse events
- Hospitalization
- Preservation of residual renal function
- Body weight
- Peritoneal small solute clearance (peritoneal creatinine clearance, peritoneal urea clearance)
- Carbohydrate absorption
- Glycemic in control in diabetic patients
- Insulin resistance in non-diabetic patients
- Long dwell sodium removal/serum sodium concentration
- Middle-molecule clearance
- Peritoneal membrane glucose exposure
- Lipid level
- Plasma total cholesterol
- Fasting plasma glucose
- Triglycerides
- Inflow pain

### Risk of bias in individual studies

We will use the Cochrane risk of bias tool to evaluate all included studies for risk of bias [14]. Items will be rated as low, high, or unclear risk of bias. Independently, two reviewers (MB and KG) will assess the risk of bias of all included studies. We will assess the risk of bias at the outcome level. For each assessment, we will provide a support for judgment. Any disagreements will be resolved through discussion and consensus. If necessary, we will involve a third reviewer (DP).

### Data synthesis

Relative risks will be calculated for dichotomous outcomes and mean differences or standardized mean differences, if different scales were used, for continuous outcomes. For the analyses of the patient and technique survival, we will calculate hazard ratios applying the generic inverse-variance method or alternatively Peto odds ratio. For all measures, 95% confidence levels will be calculated.

Clinical and statistical heterogeneity between studies will be assessed by two reviewers. For the assessment of statistical heterogeneity, *I*^2^ will be calculated. In the absence of clinical heterogeneity, and in the presence of statistical heterogeneity (*I*^2^ > 50%), we will use a random-effects model. In case of no clinical or statistical heterogeneity, we will apply a fixed-effect model. We will obtain pooled estimates of treatment effect using RevMan 5 software.

Sources of clinical heterogeneity will be evaluated through subgroup analyses.

If possible, we will undertake subgroup analyses according to:

- Cause of end-stage renal disease, e.g., diabetes
- Incident versus prevalent patients
- Peritoneal membrane transport characteristics (high, high-average, low, or low-average transport status as defined by PET)
- Concentration of glucose
- Concentration of icodextrin
- Age (children, adults,)
- Duration of follow-up (e.g., 3 versus 24 months).

Where possible, we will conduct sensitivity analyses according to the influence on the results of fixed-effect model versus random-effects model assumptions and of including trials at high risk of bias. The overall risk will be considered high if any of the domains of the Cochrane risk of bias tool were judged to be at high risk of bias.

### Meta-bias(es)

If there are ten or more studies included in the meta-analysis, we will investigate publication bias using funnel plots (using RevMan 5 software) and Egger’s test (using Meta-Essentials [14]).

### Confidence in cumulative evidence

Summary of finding tables will be prepared for summarizing confidence across studies for all relevant outcomes. For grading the quality of evidence, the five GRADE domains, risk of bias, indirectness, inconsistency, imprecision, and publication bias, will be judged [15]. The quality of the body of evidence will be assessed by two reviewers (MB and KG) independently using the GRADEpro GDT software.

## Discussion

We assume that our systematic review will be more comprehensive compared to the previously published ones due to contacting the relevant pharmaceutical companies and systematically searching published and unpublished non-English studies from China, Taiwan, and Japan. Previously published meta-analyses could not discern significant differences regarding patient-relevant outcomes [8–11]. Adding previously unknown studies and newly available data to our review might increase the chance of obtaining answers to unanswered clinical questions.

## Presenting and reporting the results

This protocol adheres to the *Preferred Reporting Items for Systematic Review and Meta-Analysis-Protocols* (PRISMA-P) [16]. Any amendments to this protocol will be reflected in an update to the PROSPERO registration. The reporting in the systematic review will adhere to the Preferred Reporting Items for Systematic Reviews and Meta-Analyses (PRISMA) [17].

## Abbrevitations

APD: Automated peritoneal dialysis
CAPD: Continuous ambulatory peritoneal dialysis
CB-ISTIC: Chongqing Branch of Institute of Scientific & Technical Information of China
CCPD: Company Profiles Database
CCPD: Continuous cyclic peritoneal dialysis
CFPD: Continuous flow peritoneal dialysis
ChiCTR: China’s Clinical Trial Registry
GRADE: Grading of Recommendations Assessment, Development and Evaluation
ICTRP: International Clinical Trials Registry Platform Search Portal
IPD: Intermittent peritoneal dialysis
JPRN: Japan Registries Network
NIPD: Nocturnal intermittent peritoneal dialysis
PD: Peritoneal dialysis
PET: Peritoneal equilibration test
RCT: Randomized controlled trial
TAO: Taiwan Academic Online
TPD: Tidal peritoneal dialysis
UF: Ultrafiltration
